# Development and characterization of a CRISPR/Cas9-mediated *RAG1* knockout chicken model lacking mature B and T cells

**DOI:** 10.3389/fimmu.2022.892476

**Published:** 2022-08-11

**Authors:** Kyung Youn Lee, Hyeon Jeong Choi, Kyung Je Park, Seung Je Woo, Young Min Kim, Jae Yong Han

**Affiliations:** Department of Agricultural Biotechnology and Research Institute of Agriculture and Life Sciences, College of Agriculture and Life Sciences, Seoul National University, Seoul, South Korea

**Keywords:** immunodeficient chicken, *RAG1* knockout, avian immunology, CRISPR/Cas9, B cell receptor, embryonic stage, B cell, T cell

## Abstract

Although birds have been used historically as a model animal for immunological research, resulting in remarkable achievements, immune cell development in birds themselves has yet to be fully elucidated. In this study, we firstly generated an immunodeficient chicken model using a CRISPR/Cas9-mediated recombination activating gene 1 (*RAG1*) knockout, to investigate avian-specific immune cell development. Unlike previously reported immunoglobulin (Ig) heavy chain knockout chickens, the proportion and development of B cells in both *RAG1* **^+/-^** and *RAG1* ^-/-^ embryos were significantly impaired during B cell proliferation (embryonic day 16 to 18). Our findings indicate that, this is likely due to disordered B cell receptor (BCR)-mediated signaling and interaction of CXC motif chemokine receptor (CXCR4) with CXCL12, resulting from disrupted Ig V(D)J recombination at the embryonic stage. Histological analysis after hatching showed that, unlike wild-type (WT) and *RAG1* **^+/-^** chickens, lymphatic organs in 3-week old *RAG1* ^-/-^ chickens were severely damaged. Furthermore, relative to WT chickens, *RAG1^+/-^* and *RAG1^-/-^* birds had reduced serum Igs, fewer mature CD4^+^ and CD8^+^ T lymphocytes. Furthermore, BCR-mediated B cell activation in *RAG1* ^+/-^ chickens was insufficient, leading to decreased expression of the activation-induced deaminase (*AID*) gene, which is important for Ig gene conversion. Overall, this immunodeficient chicken model underlines the pivotal role of *RAG1* in immature B cell development, Ig gene conversion during embryonic stages, and demonstrates the dose-dependent regulatory role of *RAG1* during immune cell development. This model will provide ongoing insights for understanding chicken immune system development and applied in the fields of immunology and biomedical science.

## Introduction

Study of the chicken immune system has contributed greatly to fundamental immunological research. In the 1910s, the graft-versus-host response was first discovered using tissue engraftment in the chorioallantoic membrane of developing chick embryos (1). In the 1960s, two types of immune system were first discovered in chickens: the bursa-dependent system, which produces antibodies, and thymus-dependent cell-mediated humoral immunity (2). Subsequently, it was determined that in chickens, the bursa has functions similar to those of the bone marrow in mammalian species (3). Although the discovery of antibody-dependent and T-cell-dependent immune systems in chickens has informed numerous immunological studies, in the absence of immunodeficient chicken models the process of avian-specific immune cell development has yet to be fully elucidated, and understanding lags behind that of equivalent processes in mammalian species. Hence, development of immunodeficient chicken models will broaden our understanding of the immune system overall, and serve as a valuable research tool.

Previously, to study B cell development in chickens, B cells have been removed by bursa dissection (4), irradiation (2), or intra-embryonic administration of chemical reagents (5). Furthermore, many studies have applied retroviral gene transfer methods using the chicken DT40 B cell line to clarify the processes involved in B cell migration and development in the bursa. Some B lineage cells do not express surface IgM (sIgM), and can migrate within the bursa follicle during the bursa stage (6). CXCR4/CXCL12 signaling is critical for B cell migration to the bursa follicle, and CXCR4 is dependent on BCR signaling (7, 8). Interestingly, delivery of a V(D)J-encoded determinant-free truncated u receptor complex (Tu) into B cell precursors by retroviral gene transfer *in vivo* can support bursal follicle colonization, B cell proliferation, and Ig gene diversification (9). In addition, follicle colonization and expansion of bursa B cells can occur, even in the absence of the BCR extracellular domain and the presence of the Igα and Igβ cytoplasmic domains (10, 11). Although V(D)J-encoded determinants are not critical for embryonic bursa B cell development, production of complete IgM complexes is important for their emigration to the periphery and spread to secondary lymphoid organs in chickens after hatching, and only B cells capable of binding specific antigen can survive and develop fully (12). Recently, it was reported that Ig heavy chain J segment knockout does not affect migration to the bursa follicle in chickens, but does lead to defective mature B cell development and subsequent emigration (13). In chickens with only the Ig light chain knocked out, some B cells can develop to full maturity and generate antigen-specific IgM and IgY following immunization (14). Hence, B cell development has been analyzed using chickens with Ig heavy or light chain knockout; however, characterization of embryonic B cell stages remains inadequate.

In chicken T cells, gene rearrangements in the T cell receptor (TCR) region diversify the antigen binding repertoire; this is a process that is highly conserved in mammalian species. During T cell development in the chicken thymus, the larger TCR chains, β and δ, are generated by recombination of genomic VDJ segments, while diversity of TCR α and γ chains is generated *via* VJ recombination (15, 16). In mice and humans, it composed of approximately 20~30 subfamilies of V_α_ and V_β_, whereas in chickens, it has simple structure consisting of only two V_α_ and V_β_ subfamilies. In mice, γδ T cells were tissue-specifically distributed according to the rearrangement combination. V_γ_3 J_γ_1 C_γ_1 T cells prefer to migrate to the skin epithelium and V_γ_5 J_γ_1 C_γ_1 cells to the intestine. However, in chickens, the three V_γ_ subfamilies undergo rearrangement without preference at E10, an early stage of thymic development, and are not known to be tissue-specific (17). In addition, CD8 αβγδ T cell subunit, which is not found in mammalian species, is found in the chicken intestine (18). Compared to the developmental process of B cells, the developmental process of T cells is highly conserved to that of mammalian species, but with some differences in details. T cell development in chickens has been investigated by injecting antibodies specific for TCR subunits (TCR1, TCR2, and TCR3) into embryos to suppress TCR function, or by conducting thymectomy after hatching (19, 20); however, complete prevention of T cell development has not been achieved.

Recombination activating gene 1 (*RAG1*) is an endonuclease that initiates V(D)J recombination by specifically inducing double-strand breaks in the recombination signal sequences (RSS) of lymphocyte progenitor cells. V(D)J recombination takes place in the Ig of B cells and the TCR of T cells, conferring immune cell diversity to multiple antigens. Although the function of *RAG1* is conserved in many species, there are significant differences in the chicken Ig and TCR structures. To gain a deeper overall understanding of bird immune cell development, we used CRISPR/Cas9 genome editing to generate *RAG1*-disrupted chickens with blocked Ig and TCR recombination. The aim was to deplete mature B and T cells. Using this model, we analyzed the overall features of lymphocyte and lymphoid organ development to characterize the chicken immune system during embryonic development and post-hatching.

## Materials and methods

### Experimental animals and animal care

The management and experimental use of chickens were approved by the Institute of Laboratory Animal Resources, Seoul National University, South Korea (SNU-190401-1-2). All experimental animals including White Leghorn (WL) and Korean Ogye (KO), were cared for according to a standard management program at the University Animal Farm, Seoul National University. The procedures for animal management, testcross analysis, and embryo manipulation adhered to the standard operating protocols of our laboratory (21).

### Construction of CRISPR/Cas9 plasmid and donor plasmid

The CRISPR kit used for constructing multiplex CRISPR/Cas9 plasmids was provided by Takashi Yamamoto (1000000054; Addgene, Watertown, MA, USA). For insertion of guide RNA (gRNA) sequences into the CRISPR/Cas9 plasmids, we designed sense and antisense oligonucleotides (**Supplementary Table 1**) and synthesized by Bionics (Seoul, Korea). The annealing of each oligonucleotides was carried out under the following thermocycling conditions; 30 s at 95°C, 2 min at 72°C, 2 min at 37°C, and 2 min at 25°C. For targeted *RAG1* gene disruption, the neomycin resistance gene with thymidine kinase promoter and a red fluorescence protein (tdTomato) gene with a cytomegalovirus (CMV) promoter were cloned into the pGEM-T Easy vector (Promega, Madison, WI, USA) using donor plasmid specific primers (**Supplementary Table 1**) containing gRNA recognition sequences.

### Culture and transfection of chicken PGCs

White Leghorn (WL) cultured PGCs used for genome editing in the present study were originally retrieved from the male gonads at embryonic day 6 by magnetic-activated cell sorting (MACS) method (22), and sub-passaged in knockout DMEM (Thermo Fisher Scientific, Waltham, MA, United States) supplemented with 20% FBS, 2% chicken serum (Millipore Sigma, Burlington, MA, USA), 1x nucleosides (Millipore Sigma), 1x nonessential amino acids, 1x ABAM, β-mercaptoethanol, 10 mM sodium pyruvate, 2 mM L-glutamine, and human basic fibroblast growth factor (10 ng/ml; Millipore Sigma). Chicken PGCs were incubated at 37°C with an atmosphere of 5% CO_2_ and 60-70% relative humidity. The PGCs were sub-passaged 5-6 days onto mitomycin-inactivated mouse embryonic fibroblasts and did not undergo any enzyme treatment. To establish *RAG1* knockout chicken PGCs, 6 μl of Lipofectamine 2000 reagent, 2 μg of CRISPR plasmids and 2 μg of donor plasmid were suspended in 1 ml Opti-MEM (Thermo Fisher Scientific) and this mixture was applied to 1 x 10^5^ cultured PGCs. Gentle pipetting was carried out at 1 h intervals and changed to PGC culture medium 4 h after transfection. G418 (300μg/ml) was added to PGC culture medium 24 h after transfection and selection was performed up to 7 days.

### Integration PCR and sequencing analysis

After treatment with CRISPR and donor plasmids, genomic DNA was extracted from part of the PGCs that were selected for puromycin. To identify *RAG1* disrupted region, genomic DNA was analyzed using knock-in PCR analysis. For sequencing analysis, the PCR amplicons were cloned into the pGEM-T easy vector (Promega) and sequenced using an ABI Prism 3030XL DNA Analyzer (Thermo Fisher Scientific). The sequence was compared against assembled genomes using the Basic Local Alignment Search Tool (BLAST).

### Production of *RAG1* knockout chickens

To produce *RAG1* knockout chickens, more than 3,000 genome-edited WL PGCs were microinjected into the dorsal aorta of KO recipient embryos (*i/i*) of Hamburger Hamilton (HH) stage 14-17 through a small window on the incubated eggs. After sealing the egg window with paraffin film, the eggs of recipient embryos were incubated until hatching after one round of candling. The hatched chicks were raised until sexual maturation, and the recipient KO roosters which were positive in WL-specific PCRs in their sperm were used for testcross with wild type (WT) hens (*I/I*) of WL. Germline chimeric chickens were identified based on feather and leg color patterns of offspring (*I/I* or *I/i*) and the individual donor PGC-derived chickens (*I/I*) were analyzed by subsequent genomic DNA integration PCR analysis for *RAG1* genome-edited chickens. Genomic DNA was isolated from individual chicken feather pulp, and genomic region containing the endogenous *RAG1* locus and donor plasmids were analyzed using specific primer sets (**Supplementary Table 1**).

### Prediction of putative off-target sites and analysis

The CRISPR RGEN tools provided Cas-OFFinder (http://www.regenome.net/cas-offinder/ *)* was used to predict nine putative off-target sites. Potential off-target sites were with up to 1-bp DNA or RNA bulge + up to 2-bp mismatches in the chicken reference genome Gallus_gallus-6.0. Each off-target site was amplified by target specific primers (**Supplementary Table 1**) and analyzed using an ABI Prism 3730XL DNA Analyzer (Thermo Fisher Scientific).

### RT-quantitative PCR analysis

Total RNAs from various embryonic stages of WT spleen, bursa and thymus along with WT, *RAG1^+/-^* and *RAG1^-/-^* bursa were isolated using Trizol Reagent (Thermo Fisher Scientific) and reverse-transcribed using the Superscript III First-strand Synthesis System (Thermo Fisher Scientific). The cDNAs of above-mentioned samples were amplified with the *RAG1* specific primers by RT-quantitative PCR (RT-qPCR). The cDNAs of embryonic day 16 (E16), embryonic day 18 (E18)- and 3-week bursa samples were amplified with the RT-qPCR primers of B-cell development related genes. All reactions were performed under the same conditions, containing 100 ng cDNA, 20× EVA green (Biotium, Hayward, CA, United States), 10× PCR buffer, 10 mM each of dNTP, 10 pM of each primer, and 0.5 U *Taq* polymerase (Bionics). RT-qPCR conditions were as follows: 95°C for 3 min, followed by 40 cycles of 95°C for 30 s, 61°C for 30 s, and 72°C for 30 s. Melting-curve profiles were analyzed for all amplicons. Each test sample was run in triplicate. Relative quantification of the target gene expression was normalized by housekeeping gene *GAPDH* and B cell specific gene *BAFF* for control. The oligonucleotides used for RT-qPCR are listed in **Supplementary Table 2**. The formula we used is as follows: 2 ^- ((Ct^_GOI_^– Ct^ *_GAPDH_* ^) - (Ct^ *_BAFF_* ^- Ct^ *_GAPDH_* ^))^.

### Histological analysis and immunohistochemistry

WT, *RAG1 ^+/-^* and *RAG1^-/-^* chickens embryonic bursa and spleen at E16 and E18 (n>3), and bursa and thymus of at 1-week and 3-weeks (n>3) were fixed in 4% paraformaldehyde (PFA) at 4°C overnight. Tissues were dehydrated in 30%, 50%, 70% and 100% ethanol, made transparent in xylene, and embedded in paraffin. Paraffin-embedded tissue blocks were obtained, sections (5 μm thick), which were deparaffinized and rehydrated for histological analysis. The sectioned samples were stained with hematoxylin and eosin and dehydrated through graded ethanol solutions followed by a xylene-based mounting solution. Also, for embryonic bursa, sectioned samples were incubated at 4°C overnight with primary antibodies, mouse anti-chicken Bu-1 FITC (8395-02), mouse anti-chicken IgM BIOT (8310-08) and mouse anti-desmin (MA5-13259, Thermo Fisher Scientific). After washing 3 times with PBS, sections were incubated with fluorescence-conjugated secondary antibodies (Alexa Fluor 488; Thermo Fisher Scientific) for 2 h at room temperature. After washing 3 times with PBS, sections were mounted with 4;6-diamidino-2-phenylindole (DAPI, Vector Laboratories, CA, USA) and visualized using microscopy (Carl Zeiss Microscopy LLC, NY, USA).

### PCR and sequencing of Ig heavy chain and light chain

Peripheral blood mononuclear cells (PBMCs) were isolated by Ficoll (Sigma Aldrich) density gradient centrifugation from EDTA-treated blood according to the manufacturer’s instructions. The rearranged chicken heavy chain and light chain was amplified from isolated PBMCs genomic DNA by target specific primers (**Supplementary Table 1**) and analyzed using an ABI Prism 3730XL DNA Analyzer (Thermo Fisher Scientific).

### Immunoglobulin quantification

Blood was collected from the 1-week, and 3-weeks of WT, *RAG1 ^+/-^* and *RAG1^-/-^* chickens (n = 4 each). The blood samples were placed for 2 h at room temperature and centrifugation for 20 min at 2,000×g. The collected serum was used for immunoglobulin quantification immediately or aliquoted and stored at -20°C for later use. Then, the serum levels of IgM (ab157692), IgY (ab157693), and IgA (ab157691) were quantified by ELISA using a chicken immunoglobulin ELISA kit (Abcam, Cambridge, UK).

### Flow cytometry analysis

Right after sacrificing the chicks, single-cell suspension from thymus and bursa were prepared to keep the samples fresh and viable. The tissues were chopped and dissociated with 0.05% trypsin-EDTA. After dissociation, cells were treated with ACK lysis buffer (A10492, Gibco, Grand Island, NY, USA) for 5 min to remove red blood cells. Cells (1 × 10^6^) were stained for 40 min on ice using the following antibodies: mouse anti-chicken Bu-1 FITC (8395-02), mouse anti-chicken IgM BIOT (8310-08), mouse anti-chicken CD45 SPRD (8270-13), mouse anti-chicken CD3 FITC (8200-02), mouse anti-chicken CD4 Alexa Fluor 647 (8201-31), mouse anti-chicken CD8α BIOT (8405-08), mouse IgG1-FITC (0102-02), mouse IgM-SPRD (0101-13), mouse IgG2b-BIOT (0104-08) and mouse IgG1-Alexa Fluor 647 (0102-31) were purchased from Southern Biotech (Birmingham, AL, USA). After washed with PBS, BIOT cells were incubated with Bv421 conjugated secondary antibodies (BD sciences, 563259) for 20 min on ice. The cells were analyzed with FACSCalibur (BD Biosciences, San Jose, CA, USA), and subsequent analyses were performed using FlowJo software (Treestar, Ashland, OR, USA). Live and dead cells were distinguished through Live/Dead Fixable Near-IR Dead Cell Stain Kit (L10119, Thermo fisher Scientific), tdtomato expressing cells were used an fluorescence minus one (FMO) controls, and the gating strategy was shown in supplementary materials (see **Supplementary Figures 6, 7**).

### MACS cell separation

MACS was performed according to the manufacturer’s instructions (Miltenyl Biotec Inc., San Diego, CA). Bursa from 3-week-old chickens were sampled and dissociated, and only B cells were sorted. A primary antibody against chicken Bu-1 (8395-02) was used on the dissociated bursa cells, and then anti-mouse IgG1 microbeads (Miltenyl Biotec, 130-048-401) were used and B cells were isolated through MACS column.

### Statistical analysis

Statistical analysis was performed using the GraphPad Prism (GraphPad Software, La Jolla, CA, USA). Significant differences among the groups were evaluated by student t-test and one-way ANOVA. A value of *P*<0.05 indicated statistical significance.

## Results

### Generation of *RAG1* knockout chickens

**Figure 1:**
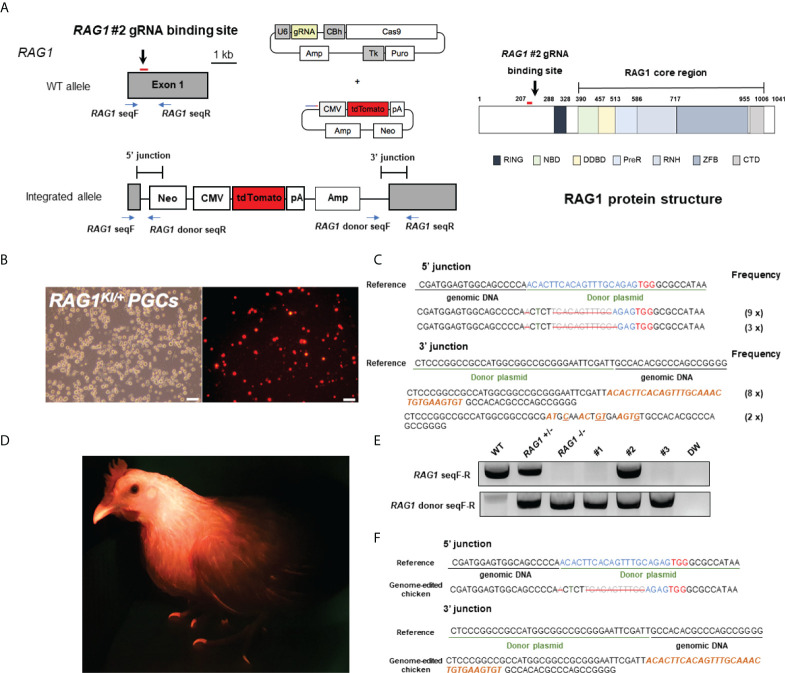
Generation of *RAG1* knockout chickens *via* primordial germ cell (PGC)-mediated germline transmission. **(A)** CRISPR/Cas9 targeting exon 1 of *RAG1* (*RAG1* #2) with a donor plasmid containing tdTomato, driven by the CMV promoter to disrupt *RAG1* expression and schematic structure of chicken RAG1 protein. In the schematic diagram of the RAG1 protein, numbers indicate amino acids number. **(B)** Expression of tdTomato in chicken PGCs. Scale bars = 100μm. **(C)** Genomic DNA analysis of targeted genome integration of 5’ and 3’ junctions in chicken PGCs using integration-specific primer sets, and sequencing results following TA cloning of amplicon. **(D)** Production of *RAG1* knockout chickens, distinguished by red fluorescence. **(E, F)** Representative results of integration-specific PCR amplification of genomic DNA from WT, *RAG1* ^+/-^ and *RAG1* ^-/-^ progeny, and results of sequencing the 5’ and 3’ junctions.

To generate *RAG1* knockout chickens, we applied the CRISPR/Cas9 mediated non-homologous end-joining genome editing method, as previously reported (21). By introducing a *RAG1* targeting CRISPR/Cas9 plasmid and donor plasmid containing *RAG1* sgRNA #2 into chicken cells, catalytic core region of *RAG1* expression was blocked by targeted integration of the donor plasmid (**Figure 1A**). After transfection with the CRISPR/Cas9 and tdTomato-containing donor plasmids, chicken primordial germ cells (PGCs) expressed tdTomato (**Figure 1B**). To characterize the targeted genome integration, we conducted sequencing analysis of the 5’ and 3’ junction regions containing the endogenous *RAG1* gene and donor plasmid in PGCs, which revealed targeted genome integration by partial deletion of nucleotides at the 5’ junction, and insertion with partial nucleotide modification at the 3’ junction (**Figure 1C**); *RAG1* knockout chickens were distinguishable by tdTomato expression (**Figure 1D**). Two germline chimeras generated 12.2% (59/480) and 3.4% (5/148) of *RAG1* knockout chickens (**Table 1**). In addition, *RAG1* knockout in chicks was confirmed by PCR amplification using knock-in specific primers, and both *RAG1* heterozygous (*RAG1^+/-^*) and homozygous (*RAG1^-/-^*) knockout chickens were detected (**Figure 1E**). Sequencing analysis of *RAG1* knockout chickens showed that the genomic modification pattern was identical to that in PGCs (**Figure 1F**). Furthermore, we evaluated potential off-target effects in *RAG1* knockout chickens using the TA cloning method. In total, six putative off-target sites were analyzed, and no off-target effects have been detected at the predicted off-target sides that have been analyzed (**Table 2**, see **Supplementary Figure 1**).

**Table 1:** Efficiency of genome-edited chickens.

Germline chimera ID	No. of hatched chicks	No. of genome-edited chicks (%)^a^
3108	480	59 (12.2)
3110	148	5 (3.4)

a Number of genome-modified chicks among hatched chicks.

**Table 2:** Potential off-target analysis in the *RAG1* knockout chicken.

Gene	Potential off-target site		Number of mismatches	Position	Bulge size (DNA or RNA)	Frequencyof off-target^a^
*RAG1*		AC **T** AtTTCACAGTcTGCAGAG**GGG**	2	Chr1 + 94848720	1 (DNA)	0/8
		ACACTTCtCAGTcTGCA **T** GAG**GGG**	2	Chr2 - 3394164	1 (DNA)	0/8
		t-ACTTCACAGTTTGCAGtG**AGG**	2	Chr2 + 37783755	1 (RNA)	0/8
		AtAC-TCACAGTTaGCAGAG**AGG**	2	Chr1 - 105840605	1 (RNA)	0/8
		ACtCTTCACAGTTTGCAcAG**TGG**	2	Chr4 + 25271271	0	0/8
		ACACT **G** TCACAcTTTGgAGAG**AGG**	2	Chr2 + 67460293	1 (DNA)	0/10

Blue uppercase letters indicate PAM sequences, lowercase letters indicate mismatch nucleotides, uppercase letters are identical nucleotides to the target gRNA, dash indicates RNA bulge, and bold underlined letters indicate DNA bulge.

a The number of off-target clones/the sequenced clones.

### *RAG1* expression profile and incomplete B cell migration in *RAG1* knockout embryonic bursa

**Figure 2:**
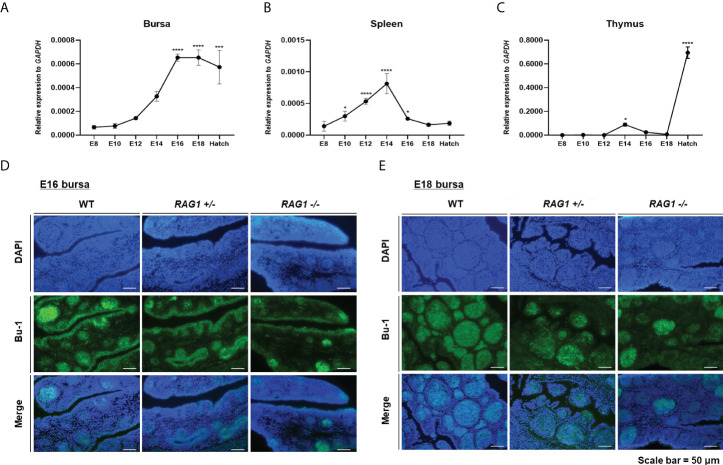
Expression profile of *RAG1* in lymphoid organs and decreased B cell population in *RAG1* ^+/-^ and *RAG1* ^-/-^ embryonic bursa. Validation of *RAG1* mRNA expression by RT-qPCR analyses in the primary lymphoid organs [bursa **(A)** and thymus **(C)**], and the secondary lymphoid organ [spleen **(B)**]. Relative expression of *RAG1* was calculated after normalization to *GAPDH* and E8 control samples, and the significance of differences among groups was evaluated by one-way ANOVA; *P < 0.05, ***P < 0.001, ****P < 0.0001. Expression of the B cell lineage marker (Bu-1) in E16 **(D)** and E18 **(E)** embryonic bursa was examined by immunohistochemistry.

To evaluate *RAG1* expression profiles during embryonic development in chicken lymphoid organs, we performed RT-qPCR in the primary lymphoid organs, the bursa and thymus and the secondary lymphoid organ spleen. *RAG1* expression in the bursa was low during early embryonic development, increased progressively to embryonic day 14 (E14), and then rose significantly from E16 to hatching, when B cells colonized and proliferated in the bursa (**Figure 2A**). By contrast, *RAG1* expression levels in the spleen increased gradually from E10, peaked at E14, and decreased again until hatching (**Figure 2B**).

T cell progenitor cells colonize the thymus epithelium in three distinctive waves: the first wave starts at E6, the second around E12, and the third wave starts between E18 and hatching (23). *RAG1* expression in the thymus was very low during early embryonic development, increasing slightly at E14 after the second wave, and subsequently rose significantly during the third wave, between E18 and hatching (**Figure 2C**). Hence, analysis of expression profiles in lymphoid organs showed that *RAG1* levels vary with B and T cell developmental stage.

The bursa is a unique primary lymphoid organ in birds that distinguishes it from other species; therefore, we next investigated whether embryonic B cell development is impaired in the *RAG1* knockout chicken bursa. First, we performed immunostaining of the B cell lineage marker, Bu-1, in E16 and E18 bursa samples, representing the periods when B cells actively proliferate. Embryonic bursa follicles developed in E16 wild-type (WT), *RAG1^+/-^*, and *RAG1^-/-^* embryos, with varying degrees of Bu-1-positive cells present in lymphoid follicles and follicle-associated epithelium regions (**Figure 2D**). In E18 embryos, most Bu-1-positive cells were located in lymphoid follicles in WT bursa, whereas in *RAG1^+/-^* and *RAG1^-/-^* embryos, there was a slight decrease in Bu-1-positive cells in lymphoid follicles (**Figure 2E**). These results demonstrate that *RAG1* is involved in embryonic B cell development in chickens.

### *RAG1* is critical for BCR-mediated signaling during embryonic B cell development

**Figure 3:**
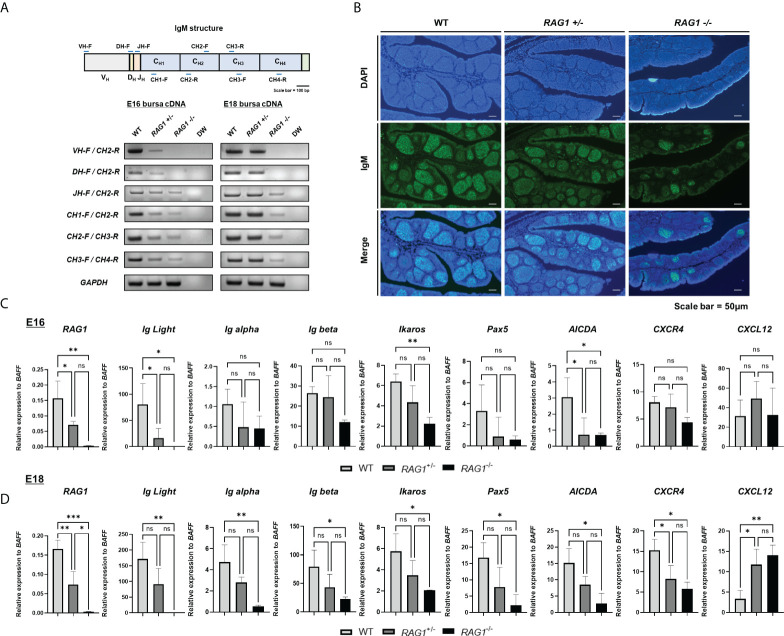
*RAG1* deficiency impairs B cell development in embryonic bursa, as demonstrated by reduced sIgM expression and impaired *CXCR4* and *CXCL12* interaction in *RAG1* ^+/-^ and *RAG1* ^-/-^ embryonic bursa. **(A)** Expression of each immunoglobulin M domain in total bursa cDNA samples from each group, as determined by RT-PCR. **(B)** The proportions of surface IgM (sIgM) -positive cells in E18 bursa from the WT, *RAG1* ^+/-^, and *RAG1* ^-/-^ groups were determined by immunostaining with IgM. Relative expression levels of *RAG1*, immunoglobulin light chain (Ig Light), B cell development-related genes, and bursa environment-related genes were calculated after normalization to *GAPDH* and B cell specific gene *BAFF* at E16 **(C)** and E18 **(D)**, and the significance of differences among groups was determined by one-way ANOVA; *P < 0.05, **P < 0.01, ***P < 0.001, ns, no significance.

Since *RAG1* mediates immunoglobulin (Ig) V(D)J recombination, RNA was isolated from E16 and E18 WT, *RAG1^+/-^*, and *RAG1^-/-^* embryo bursa and RT-PCR were performed to amplify each IgM fragment and determine which segments were fully rearranged. At E16, expression of all IgM segments was weak in *RAG1^+/-^* bursa relative to that in WT samples, while *RAG1^-/-^* samples showed faint bands for the J segment and constant region, with no bands detected from the VD or DJ junctions. At E18, amplicons for all IgM segments were amplified from *RAG1^+/-^* bursa; however, *RAG1^-/-^* embryos continued to express very low levels of these molecules (**Figure 3A**). During chicken embryonic development, B cells can develop in the absence of V(D)J determinant domains, whereas B cell receptor (BCR) mediated signaling is critical (11); therefore, we analyzed the population of sIgM-positive cells in E18 embryonic bursa by immunohistochemistry. The results showed that the proportions of B cells expressing sIgM were decreased in *RAG1^+/-^* and *RAG1^-/-^* than in WT embryos (**Figure 3B**).

Since expression of sIgM, an important part of the BCR complex, was disrupted in *RAG1^+/-^* and *RAG1^-/-^* relative to WT embryos, we next conducted RT-qPCR of E16 and E18 bursa cDNA to evaluate expression of several genes related to B cell development; the idea was to further determine the role of *RAG1* in chicken bursa B cell development. To validate the incomplete B cell development not simply due to the absence of B cells, we normalized the relevant gene expression to the B cell specific gene in chicken, B cell-activating factor of the tumor necrosis factor family (*BAFF*) (see **Supplementary Figure 2**). During embryonic development, *RAG1* and immunoglobulin light chain (IgL) expression levels were low in *RAG1^+/-^* and almost absent in *RAG1^-/-^* embryos. Moreover, levels of the B cell development related gene *Ikaros* (which are critical regulators of BCR signaling) and the gene conversion related gene, *AICDA*, were significantly lower in *RAG1^-/-^* than in WT embryos at E16 and E18. Whereas, BCR complex components *Ig alpha*, *Ig beta*, and *Pax5* (which is involved in B cell differentiation) were significantly lower in *RAG1^-/-^* than in WT embryos at E18 (**Figures 3C, D**). Additionally, B cell proliferation and migration in E16–E18 bursa proceeds *via* interaction between *CXCR4*-expressing B cells and the chemokine, *CXCL12*, which is secreted from bursa mesenchymal cells. Compared with WT embryos, expression of *CXCR4* in bursa from *RAG1^+/-^* and *RAG1^-/-^* embryos was reduced significantly, whereas that of *CXCL12* was increased significantly in *RAG1^-/-^* embryos at E18 (**Figure 3D**). These results indicate that the period (E16-E18) when immature B cell migrate into the bursa and for development, BCR-mediated signaling influences B cell development, and that interaction between *CXCR4* and *CXCL12* above a certain threshold is important for development and differentiation of progenitor B cells in the chicken embryonic bursa.

**Figure 4:**
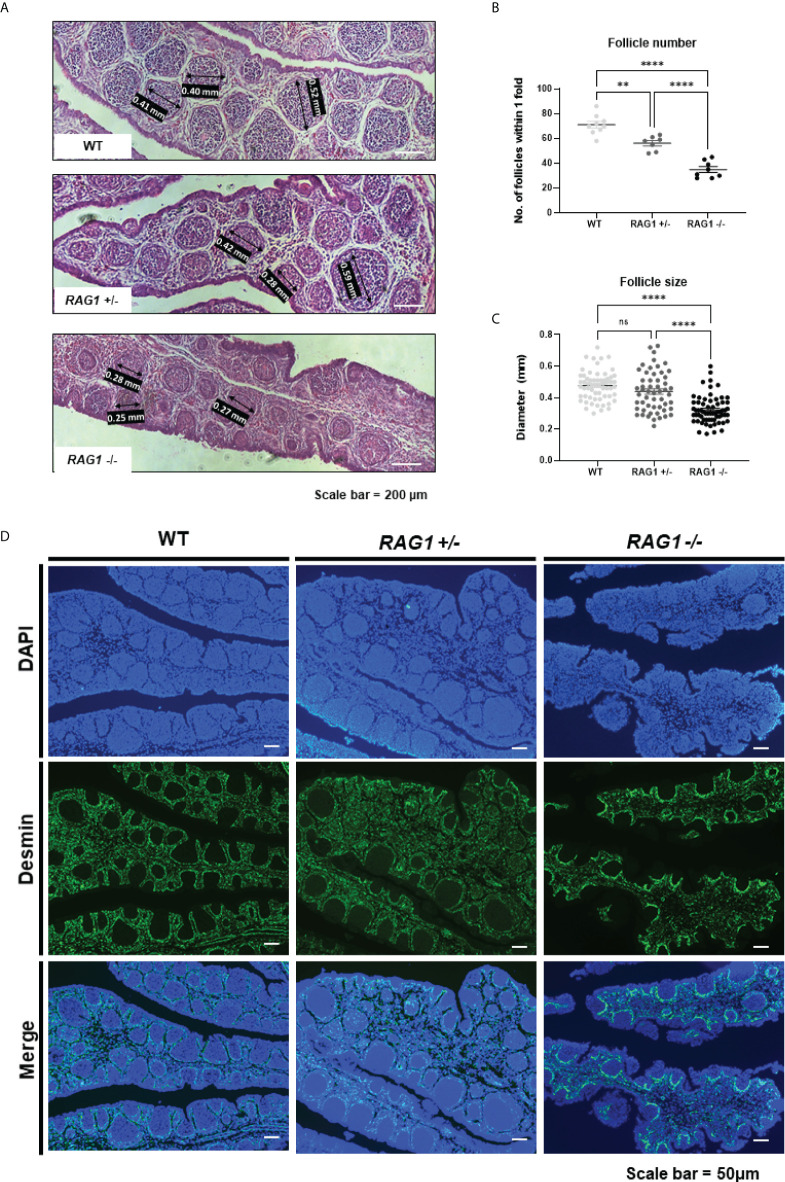
Impaired development of embryonic B cell bursa follicle. **(A)** Histological images of WT, *RAG1* ^+/-^, and *RAG1* ^-/-^ E18 bursa. The number of follicles **(B)** and diameter **(C)** of bursa follicles within 1-fold of bursa. The significance of differences among groups was calculated by one-way ANOVA; ns, no significance, **P < 0.01 and ****P < 0.0001. **(D)** Embryonic bursa sections were stained with desmin at E18 to identify bursa follicle compartmentalization.

Compared to WT, the development of B cells in the bursa is not complete in *RAG1^+/-^* and *RAG1^-/-^* embryos, but B cells are still present. In order to analyze B cell follicle formation in late embryonic B cell stage, we analyzed the numbers and diameters of the bursa follicles within 1-fold in the E18 bursa. Through histological staining, we identified that bursa follicles are sparsely present in *RAG1^-/-^* bursa (**Figure 4A**). The number of follicles within 1-fold was significantly decreased in *RAG1^+/-^* (56.14 ± 5.79, n = 7) and *RAG1^-/-^* (34.88 ± 6.75, n = 8) compared to WT (71.11 ± 7.96, n = 9) (**Figure 4B**). The follicle size was significantly reduced in *RAG1^-/-^* (0.32 ± 0.09 mm, n = 64) compared to WT (0.48 ± 0.09 mm, n = 61) and *RAG1^+/-^* (0.44 ± 0.13 mm, n = 54) (**Figure 4C**). In addition, when the separate layer surrounding the B cell follicle was stained with desmin, it was confirmed that follicle compartmentalization was incomplete in *RAG1^+/-^* and *RAG1^-/-^* embryos compared to WT controls (**Figure 4D**).

### Impaired development of lymphoid organs, and absence of Ig recombination in *RAG1* knockout chickens

**Figure 5:**
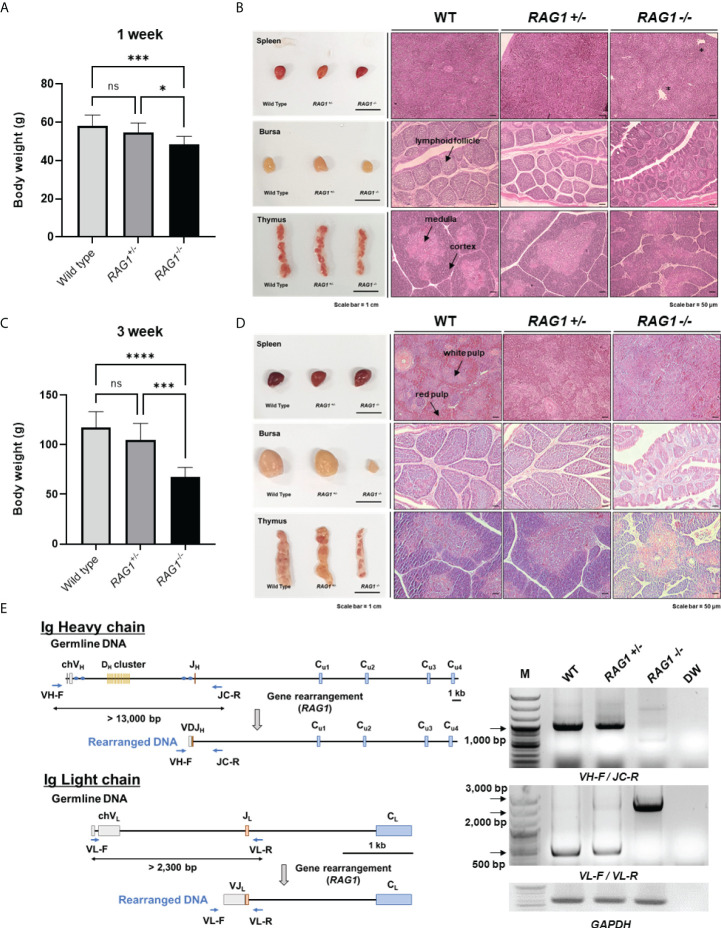
Impaired development of lymphoid organs in *RAG1* knockout chickens. Comparison of WT, *RAG1* ^+/-^, and *RAG1* ^-/-^ chicken body weight at 1 week **(A)** and 3 weeks **(C)**. The significance of differences among groups was calculated by one-way ANOVA; *P < 0.05, ***P < 0.001, ****P < 0.0001. Morphology and histological images of WT, *RAG1* ^+/-^, and *RAG1* ^-/-^ chicken lymphoid organs at 1 week **(B)** and 3 weeks **(D)**. Asterisks indicate gross lesions. Prepared sections were stained with hematoxylin and eosin (H&E). Scale bars = 1 cm for bright field images, and 50 μm for H&E-stained images. **(E)** Diagram of immunoglobulin heavy chain (top) and light chain (bottom) loci in germline and rearranged DNA. PCR analysis of PBMC gDNA from 1-week old WT, *RAG1* ^+/-^, and *RAG1* ^-/-^ chickens using specific primers (VH-F_JC-R and VL-F_VL-R) targeting rearranged immunoglobulin DNA. ns, no significance.

Next, we examined *RAG1* knockout chickens for developmental defects after hatching. The mean body weight of 1-week-old *RAG1^-/-^* chickens was significantly lower (48.63 ± 4.07 g, n = 8) than that of *RAG1^+/-^* (54.83 ± 4.83 g, n = 18) or WT (58.27 ± 5.48 g, n = 11) birds (**Figure 5A**). Phenotypically, there was no significant difference among the three groups with respect to the morphology of lymphoid organs in 1-week-old chickens. Histological analysis of lymphoid organs also revealed no significant differences between *RAG1^+/-^* and WT chickens; however, slight gross lesions of the spleen were observed in *RAG1^-/-^* chickens, with incomplete bursa follicle development and no clear boundary between the thymus cortex and medulla (**Figure 5B**).

In 3-week-old chickens, mean body weight differed more significantly among groups than that of 1-week-old birds. Body weight of chicks in the WT, *RAG1^+/-^*, and *RAG1^-/-^* groups was 117.38 ± 15.81g (n = 8), 104.92 ± 16.50 g (n = 13), and only 67.67 ± 9.44 g (n = 6), respectively (**Figure 5C**). In addition, undersized bursa and thymus were observed in *RAG1^-/-^* chickens, and histological analysis showed severe defects of all lymphoid organs (**Figure 5D**). These results reveal severe damage to lymphoid organs, resulting from incomplete immune cell development in *RAG1^-/-^* chickens.

In addition, genomic DNA was isolated from peripheral blood mononuclear cells (PBMCs) of 1-week-old WT, *RAG1^+/-^*, and *RAG1^-/-^* chickens, and PCR targeting the rearranged immunoglobulin heavy and light chains was performed. In germline DNA, the distance between the V and J segments at the Ig heavy chain locus is > 13,000 bp, while after recombination it reduces to approximately 1,000 bp, which can be amplified by PCR using specific primers. Bands of around 1,000 bp were observed in WT and *RAG1^+/-^* samples, whereas no band was amplified from *RAG1^-/-^* chicken DNA. In the case of the light chain locus, a band of approximately 500 bp, indicating rearrangement, was observed in WT and *RAG1^+/-^* chicken DNA, whereas amplification of germline genomic DNA from *RAG1^-/-^* chickens generated amplicons between 2,000 bp and 3,000 bp (**Figure 5E**).

### Reduction of mature B and T cell numbers in *RAG1* knockout chickens

**Figure 6:**
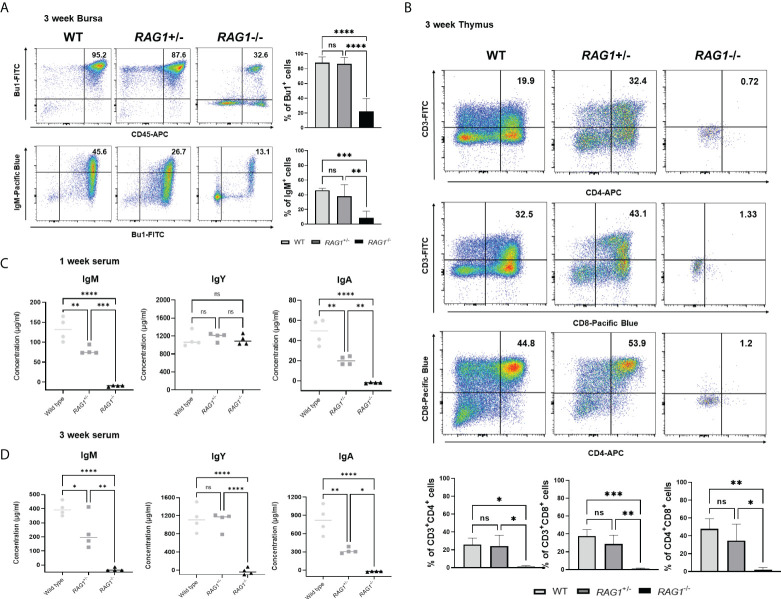
Immune cell populations and quantification of secreted immunoglobulins in *RAG1* knockout chickens. Representative flow cytometry analysis of B and T cell subpopulations in the bursa **(A)**, and thymus **(B)** of 3-week-old chickens. Statistical analysis was performed on Bu-1 and IgM positive cells in the bursa as well as CD4^+^ and CD8^+^ single-positive (SP) T cells, and the CD4^+^CD8^+^ double-positive (DP) T cell lymphocyte subpopulation in the thymus. Serum IgM, IgY, and IgA levels in 1-week **(C)** and 3-week **(D)** -old WT, *RAG1* ^+/-^, and *RAG1* ^-/-^ chickens, as determined by ELISA. Each dot represents an individual chicken. The significance of differences among groups was assessed by one-way ANOVA; *P < 0.05, **P < 0.01, ***P < 0.001, ****P < 0.0001, ns, no significance.

**Figure 7:**
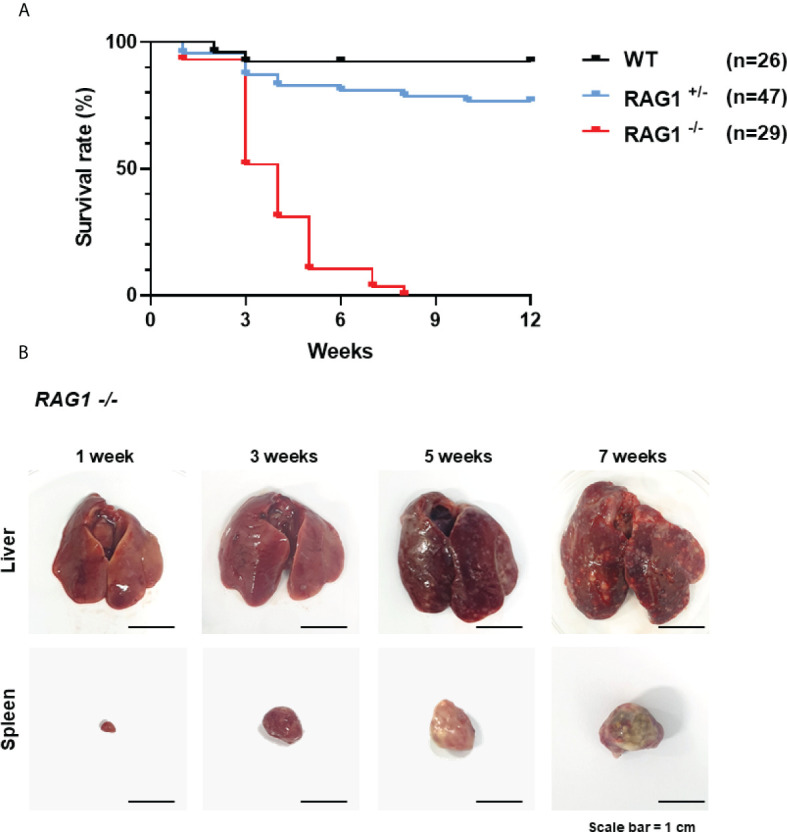
Survival rate of WT, *RAG1* ^+/-^, and *RAG1* ^-/-^ chickens in conventional breeding condition. **(A)** A curve showing the survival rate after hatch. **(B)** The disrupted organs (liver and spleen) of *RAG1* ^-/-^ chickens over time.

Next, we analyzed the immune cell subpopulations of B cells in the bursa of 3-week-old WT, *RAG1^+/-^*, and *RAG1^-/-^* chickens by flow cytometry analysis. In 3-week-old *RAG1^-/-^* chicken bursa, a minority of the cell population was positive for Bu-1; however, this population was very small relative to that in WT and *RAG1^+/-^* chickens (**Figure 6A**). Also, we analyzed the T cell population in the thymus. Our results showed that there was no significant difference in the immature CD4^+^CD8^+^ double-positive cell population and mature CD4^+^ and CD8^+^ single-positive cell populations between WT and *RAG1^+/-^* chickens, whereas these cells were almost absent in *RAG1^-/-^* chickens. (**Figure 6B**). We also compared serum immunoglobulin levels among 1- and 3-week-old WT, *RAG1^+/-^*, and *RAG1^-/-^* chickens. Serum IgM and IgA levels were significantly lower in *RAG1^+/-^* and *RAG1^-/-^* chickens than in WT controls at both 1 and 3 weeks. IgY-producing B cells are generated by class switching from the IgM-producing B-cell precursors (24), and despite the absence of serum IgM in *RAG1^-/-^* chickens, there is no significant difference in IgY between groups for up to 1 week (**Figure 6C**). These results suggest the presence of maternally transmitted IgY in *RAG1^-/-^* chickens within 1 week. However, maternal IgY was no longer present at 3 weeks in *RAG1^-/-^* chickens (**Figure 6D**). Our results confirmed that mature B and T cell differentiation was incomplete in *RAG1^+/-^* and *RAG1^-/-^* chickens, resulting in decreased serum immunoglobulin production and lacks of mature T cells in thymus. When *RAG1^-/-^* chickens were raised under normal breeding conditions, inflammatory reaction occurs in the liver and spleen from 3-week after hatching, with chickens surviving for a maximum of 8 weeks (**Table 3** and **Figure 7A, B**).

**Table 3:** Testcross analysis of *RAG1* heterozygous chicks.

No. of hatched chicks	No. of WT (%)	No. of heterozygous (%)	No. of homozygous (%)
77	18 (23.4)	37 (48.1)	22 (28.5)

### Regulation of B cell development and antibody diversification by *RAG1* after hatch

**Figure 8:**
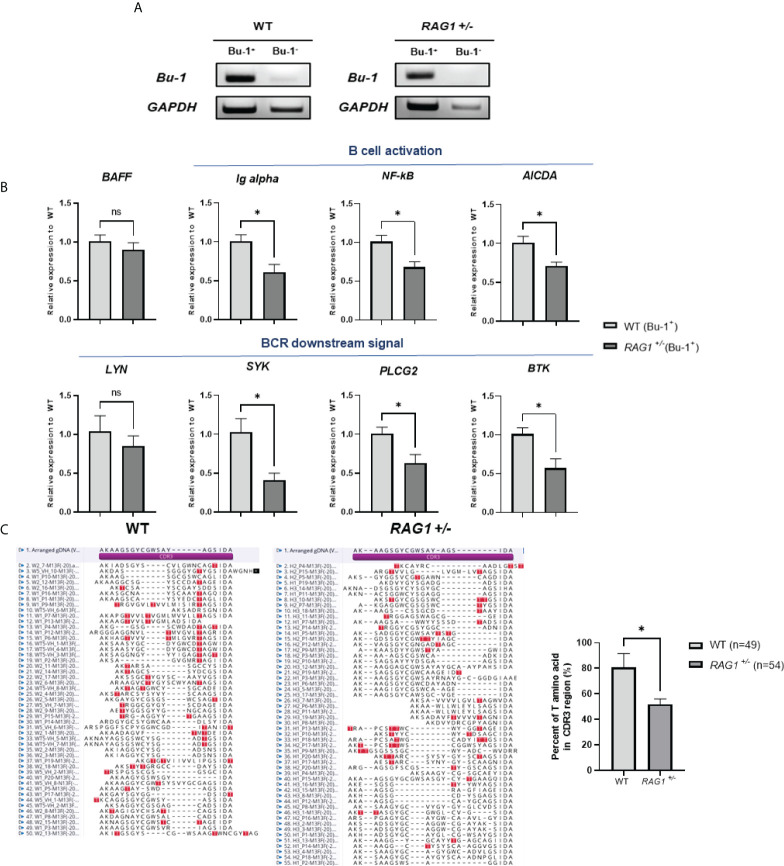
Immature B cell development caused by *RAG1* haploinsufficiency affects immunoglobulin gene conversion. **(A)** Verification of *Bu-1* and *GAPDH* expression in MACS sorted Bu-1 positive cells for RT-PCR. **(B)** Relative expression of B cell development and BCR downstream signal related genes in Bu-1-positive sorted B cells from the bursa of 3-week-old WT and *RAG1* ^+/-^ chickens, calculated after normalization to *GAPDH* and WT samples. The significance of differences between groups was assessed by Student t-test; *P < 0.05. **(C)** Analysis of the proportion of clones containing threonine codons in the region encoding the CDR3 domain of the immunoglobulin heavy chain in PBMC gDNA from 3-week-old WT and RAG1^+/-^ chickens, and amino acid sequence alignment of CDR3 domains based on sequencing analysis. Red box, threonine within the CDR3 region. ns, no significance.

In *RAG1^-/-^* chickens, VDJ rearrangement did not occur and no serum IgM was present (**Figures 5E**, **6C**); therefore, we conducted further study of WT and *RAG1^+/-^* chickens. The chicken antibody diversification process is affected by gene conversion after DNA rearrangement. We sorted Bu-1-positive B cells from bursa of 3-week-old WT and *RAG1^+/-^* chickens by magnetic-activated cell sorting (MACS), isolated RNA from these cells (**Figure 8A**), and conducted RT-qPCR analysis of genes related to B cell activation and downstream of BCR activation. Relative expression level of *BAFF* (a cytokine produced by B cells) were similar, while *Ig alpha* (a component of the BCR complex), *NF-κB* (which is activated following B cell activation), and *AICDA* (which is involved in gene conversion) were significantly lower in *RAG1^+/-^* than WT Bu-1 positive B cells. Furthermore, downstream of BCR signal related genes *SYK* (which is required for B cell activation), *PLCG2* (mediator of B cell signal) and *BTK* (which is required for transmitting signals from BCR) were significantly decreased in *RAG1^+/-^* Bu-1 positive B cell (**Figure 8B**).

To further assess the completion of B cell activation in *RAG1^+/-^* chickens, as well as the expression levels of *AICDA*, which affects gene conversion, we isolated PBMCs from the blood of 3-week-old WT and *RAG1^+/-^* chickens and analyzed the VDJ region of the immunoglobulin heavy chain sequence. Threonine are not present in chicken germline immunoglobulin D cluster DNA, and are only generated by either gene conversion or somatic hypermutation during B cell development (25). Therefore, the proportion of clones having threonine in the region encoding the complementarity-determining region (CDR) 3 domain of the heavy chain was analyzed. We found that 39 of 49 sequences from WT clones had threonine in the CDR3 domain, whereas in *RAG1^+/-^* samples, only 28 of 54 clones encoded threonine (**Figure 8C**). In addition, analysis of amplified light chain sequences from 3-week-old WT, *RAG1^+/-^*, and *RAG1^-/-^* chickens revealed no rearrangement in *RAG1^-/-^*, with the CDR1 and CDR3 domain sequences identical to those of germline DNA (see **Supplementary Figure 3**). These results show that *RAG1* regulates B cell maturation and ultimately influences antibody diversification in chickens in a dose-dependent manner.

## Discussion

Historically, chickens have been valuable animal models for basic immunological studies. Genome-edited animal models lacking both mature B and T cells have been reported in various species (26–29); however, studies of the unique characteristics of the avian immune system have been limited by technical issues. To date, chickens with Ig heavy chain knockout, with functional T cells and depleted peripheral B cells (13), and with Ig light chain knockout, leading to reduced peripheral B cells and deletion of the IgM heavy chain of the CH1 domain have been reported (14); however, genome-edited chickens lacking both B and T cells have not been reported. In this study, we generated an immunodeficient chicken model that lacks both mature B and T cells, by targeting *RAG1*, thereby broadening our understanding of chicken immune system development from embryonic development to post-hatching stages.

During embryonic B cell development, pre-bursal cells migrate to the bursal follicle, and retroviral-mediated *in vivo* delivery of truncated μ chain to B cell precursors supports B cell proliferation within bursal follicles (6). In addition, there is no difference in the development of B cells in the pre-hatch bursa between WT and Ig heavy chain J segment knockout chickens; however, mature B cell development and migration to peripheral lymphoid organs is impaired, while migration of B cells to the bursa follicle at the embryonic stage is unaffected (13). Interestingly, our data demonstrate that B cell development is incomplete in both *RAG1^+/-^* and *RAG1^-/-^* chicken embryos, and that expression of Ig alpha, Ig beta, and constant regions constituting the BCR complex was significantly reduced in bursa. Prior to D_H_ to J recombination in mice, immunoglobulin germline transcripts are transcribed from the intronic enhancer (Eμ) between the J segment and the μ constant domain, instead of *via* the V_H_ promoter (30). RT-PCR analysis of bursa cDNA from *RAG1^-/-^* chickens, confirmed a lack of V and D segment transcription; however, weak transcription of the J segment and the μ constant domains was detected. Furthermore, immunohistochemistry analyses of IgM in E18 bursa confirmed that a small number of cells translated the μ constant domains. These results indicate that, functional IgM cannot be transcribed from the V_H_ promoter in the absence of recombination in *RAG1^-/-^* chickens; however, as in mice, a partial germline transcript generated *via* the intronic enhancer, remains in the embryonic bursa and can form a BCR complex. When sequencing was performed on the RT-PCR band (JH-F/CH2-R) observed in **Figure 3A**, it was confirmed that all sequences were identical to the reference sequence (see **Supplementary Figure 4**). That is, it can be seen that the constant domain is expressed from the J segment despite the very small number of cells expressing IgM. As data to support our hypothesis, it has been recently reported that the immunoglobulin constant domain is expressed in an alternative translation initiation site in mouse neurons. And the constant domain (Cμ) was also expressed in the *RAG1* knockout mouse lacking the variable immunoglobulin domain (31). Therefore, we believe that IgM positive cells observed in embryonic bursa were expressed by an uncharacterized promoter other than the canonical promoter. Although these data are insufficient to fully explain embryonic B cell development process, more in-depth analysis to prove it can be conducted in the next study. *Ikaros* is expressed throughout B cells development in mice, and *Ikaros-*deficient mice show deficiencies of both mature B cells and progenitor B cells (32). On the other hand, *Ikaros* mutant DT40 has defect in BCR signaling, affecting calcium mobilization, but is viable, indicating that *Ikaros* is critical for B cell fate decision and maintenance (33). *Ikaros* is associated with BCR signaling and contributes to B cell development in chickens, and is downregulated in *RAG1^+/-^* and *RAG1^-/-^* B cells, indicating that B cell development is disrupted at the embryonic bursa stage.

In mice, the bone marrow microenvironment is important for development of early B cells and for activation of essential receptors such as BCR and CXCR4 (34). Unlike mammalian species, the bursa microenvironment in chickens is critical, as a site of B cell development (35). The cytokine, BAFF is not expressed in mouse or human bone marrow, and is not involved in early B cell differentiation in these species. Rather, it influences the survival and maturation of peripheral B cells (36); however, in chickens, BAFF is expressed in both the bursa and peripheral lymphoid organs and contributes to immature B cell development and mature B cell survival (37). Comparisons of *BAFF* and *BAFFR* expression between the WT and *RAG1^+/-^* groups revealed, no significant difference in *BAFF* levels, while *BAFFR* expression was lower in *RAG1^+/-^* chickens, confirming insufficient B cell developmental signal transduction through BAFF. The CXCR4-CXCL12 interaction is well conserved in mice and humans, and is important for B cell development and migration; it regulates stem cell migration to the bone marrow (38), migration of immature B cells from the bone marrow to the periphery (39), and migration of differentiated plasma cells into the bone marrow (40). A recent report demonstrated that this interaction in chickens is involved in migration of pre-bursal cells to the bursa follicle, and B cell emigration to the periphery. *In vivo* treatment with CXCR4 antagonist, AMD3100, inhibits B cell migration to the bursa, as well as proliferation in the bursa follicle (7, 8). Hence, the interaction between the cytokine and B cell receptor in the bursa microenvironment is important for B cell development. In both *RAG1^+/-^* and *RAG1^-/-^* bursa, CXCR4 expression decreased, and that of CXCL12 increased. During normal B cell development, CXCR4 expression increases, while that of CXCL12 decreases, to mediate B cell migration and emigration within the bursa. We speculate that *RAG1^+/-^* and *RAG1^-/-^* chickens overexpress CXCL12 due to developmental delay, or to promote recruitment of CXCR4^+^ B cells; however, further studies using total RNA sequencing are needed to determine the exact factors underlying the incomplete development of embryonic B cells, even in *RAG1^+/-^* chickens expressing half of normal *RAG1* levels.

In addition, compared with WT chickens, those on the *RAG1^+/-^* group showed impaired B cell development during embryonic stages, as well as significantly reduced circulating IgM and IgA levels after hatching, but similar levels of IgY. In the previously reported Ig heavy chain knockout chickens, IgY levels decreased in the heterozygotes after hatching compared with WT controls, while there was no significant difference in the IgM level, a finding opposite to our results (13). These findings suggest that, in *RAG1^+/-^* chickens, the number of sIgM expressing cells among bursal B cells is low, leading to low serum IgM levels after hatching. IgY, is maternally derived, and its concentration varies depending on the physical strength and breed of chicken; hence, variation is expected. In addition, it is known that circulating endogenous IgY is maintained within 2 weeks and the offspring synthesize their own IgY from 5 days after hatching (41). Therefore, IgY observed in 1 weeks of *RAG1* ^-/-^ chicken was maternally derived, whereas 3 weeks of *RAG1* ^-/-^ chicken were absent of IgY, suggesting that offspring were unable to generate themselves. And small number of Bu-1 positive cells were detected in the 3weeks of *RAG1^-/-^* chicken bursa; however, Bu-1 is also expressed in macrophages (42), and qRT-PCR results of 3-week bursa cDNAs for macrophage marker genes, *TIMD4* and *CSF1R* showed that *CSF1R* levels were significantly increased in *RAG1* ^-/-^ chicken bursa (see **Supplementary Figure 5**). In addition, it has recently been reported that disruption of the N-terminal core domain of RAG1 in s Syrian hamster model increases the proportion of macrophages and NK cells despite decreased numbers of B and T cells in lymphatic organs. Therefore, we speculate that Bu-1 positive cells in *RAG1^-/-^* chickens were considered to be immature B cell or possibly macrophage. In chicken, IgA production requires stimulation by T cells expressing TCR2 (V_β_1), and the relatively low levels of IgA in the serum of *RAG1^+/-^* chickens are considered to be the consequence of several factors including incomplete B cell development and insufficient activation by mature T cells (19). Our results show that the proportion of mature CD4^+^ and CD8^+^ T cells is lower in *RAG1^+/-^* and *RAG1^-/-^* than in WT chickens. Compared to WT, it was confirmed that *RAG1* ^+/-^ chickens also had a defect in the mature B and T cells development, however direct comparisons between WT and *RAG1* ^-/-^ are shown for most *RAG1* knockout model animals. A number of hypomorphic mutations have been reported in *RAG1* due to reduced gene expression levels or functional activity, resulting in immune cell dysregulation (43). And when hypomorphic mutations (F971L, R972Q and R972W) were induced in the mouse model, defects occurred in early thymocyte (CD4^-^ and CD8^-^; DN stage) development, and immature B cells (pro-B and pre-B1) was increased compared to WT. Also, abnormalities of *IgH* and TCR beta repertoire affecting receptor diversity (44). Therefore, we speculated that either reduced expression levels of *RAG1* in *RAG1^+/-^* chicken, or impaired binding affinity due to truncation of catalytic core region in one allele, leading to immune cell dysregulation.

Unlike mammalian species, chickens have a high ratio of γδ T cells, which can comprise up to 50% of the circulating T cell population (45). In both mammals and chickens, the detailed functions and effector mechanisms of γδ T cells are still poorly understood, compared with those of αβ T cells. Birds have the advantage of being manipulated during embryonic development, and during embryonic development αβ T cells did not develop, but γδ T cells began to develop around embryonic day 10. In addition, innate immune cells such as dendritic cells, macrophages and NK cells began to develop around embryonic day 5~8 (46). *RAG1^-/-^* embryo do not have γδ T cells, but do have innate immune cells. Therefore, it will be interesting to study whether it can become as efficient vaccine production platform by providing a favorable environment for the virus to be efficiently amplified, when inoculating the virus for vaccine production. Also, we will examine whether the proportion of cells involved in innate immunity related cells has changed. Furthermore, it would be interesting to investigate the effector function of γδ T cells by comparing the anti-inflammatory response between WT and *RAG1^-/-^* embryos when the virus is inoculated during embryonic stage when innate immune cells are present but γδ T cells are absent. Therefore, in the future, we would like to extend insight into the innate immune cell development at pre-hatch and the interaction between the function of innate and adaptive immune cell against virus infection during embryonic development and before 3-weeks using the *RAG1* knockout chicken model developed in this study.

In chicken B cell development, there is only functional V_L_ segment and a unique J_L_ segment in the light chain, and one functional V_H_ segment with highly conserved D_H_ clusters and a unique J_H_ segment in the heavy chain locus. Thus, only limited diversity is produced after V(D)J recombination and a gene conversion mechanism occurs in the pseudo V segments are required for antibody diversification (47–49). In chickens, Ig diversification proceeds *via* the mechanism of gene conversion, and gene conversion in the DT40 cell line is dose dependently affected by expression of the *AID* gene (50). *AID* is expressed in germinal center B cells in mice and humans, and is involved in somatic hypermutation and Ig class switching (51, 52), whereas it is expressed in chicken bursa and contribute to gene conversion. During B cell development, gene conversion begins at the bursa stage, and *RAG2* is strongly expressed during the rapid expansion phase of bursa B cell development; however, *RAG2* disrupted DT40 cells can undergo Ig gene conversion (53). Our results show that *AID* expression is significantly reduced in *RAG1^+/-^* and *RAG1^-/-^* bursa at E16 and E18; therefore, to determine whether *RAG1* affects gene conversion, we analyzed the CDR3 region of the Ig heavy chain in 3-week old WT and *RAG1^+/-^* PBMC genomic DNA. Unfortunately, no rearrangement occurred in *RAG1^-/-^* chicken heavy chain sequence, therefore the region was too long for PCR amplification (>10kb) and could not be analyzed. Expression levels of the B cell development related genes, *BAFFR*, *Ig alpha*, *NF-κB* and *AID*, were significantly decreased in Bu-1^+^ cells sorted from 3-week old *RAG1^+/-^* chicken bursa. In addition, threonine is not found in the genomic D region, while threonine present in the region encoding the CDR3 domain of the rearranged V(D)J region can be generated by gene conversion or somatic hypermutation (25, 54). Our sequencing analysis showed that, the number of clones with threonine in the CDR3 region was significantly lower in *RAG1^+/-^* chickens than in WT controls. Consequently, for the development of mature B cells, an intact BCR complexes must be formed through V(D)J recombination, and gene conversion in B cells occurs through downstream signaling. And it was confirmed that gene conversion did not occur unless V(D)J recombination was performed in B cells  through *RAG1^-/-^* chickens. Overall, our data demonstrate that the development of immature B and T cells is incomplete in *RAG1^+/-^* as well as *RAG1^-/-^* model chickens, and that gene conversion is also affected, suggesting that *RAG1* functions in a dose-dependent manner.

In conclusion, we generated *RAG1* knockout genome-edited chickens for the first time, and demonstrate that they lack both mature B and T cells. Furthermore, we analyzed the overall processes of B and T cell development and differentiation in this context. This immunodeficiency model could be applied to elucidate the pathogenesis of viruses such as Marek’s disease virus, infectious bursal disease virus, and avian influenza virus. In addition, this model has potential to aid elucidation of γδ T cell function, to identify interaction between innate immunity and adaptive immunity, and to contribute to vaccinology research. Therefore, this avian immunodeficiency model is expected to contribute substantially to understanding of viral pathogenesis and to expand immunological research.

## Data availability statement

The original contributions presented in the study are included in the article/**Supplementary Material**. Further inquiries can be directed to the corresponding author.

## Ethics statement

The animal study was reviewed and approved by Institute of Laboratory Animal Resources, Seoul National University, South Korea (SNU-190401-1-2).

## Author contributions

KL and JH designed the research. KL, HC, and KP performed the experiments. SW and YK interpreted and reviewed the data. KL wrote the first draft of the manuscript. YK and JH helped to writing the final version of the manuscript. All authors contributed to the article and approved the submitted version.

## Funding

This work was supported by the National Research Foundation of Korea (NRF) grant funded by the Korea government (MSIP) (No. NRF-2015R1A3A2033826).

## Conflict of interest

The authors declare that the research was conducted in the absence of any commercial or financial relationships that could be construed as a potential conflict of interest.

## Publisher’s note

All claims expressed in this article are solely those of the authors and do not necessarily represent those of their affiliated organizations, or those of the publisher, the editors and the reviewers. Any product that may be evaluated in this article, or claim that may be made by its manufacturer, is not guaranteed or endorsed by the publisher.

## Abbreviations

Blast, Basic Local Alignment Search Tool; CRISPR, clustered regularly interspaced short palindromic repeats; Cas9, CRISPR-associated protein 9; RAG1, recombination activating gene 1; dNTP, deoxynucleotide gRNA, guide RNA; PAM, protospacer adjacent motif; PGC, primordial germ cell; WL, White Leghorn; KO, Korean Ogye; WT, wild type; BCR, B cell receptor; Ig, immunoglobulin; TCR, T cell receptor.
