# Supplementary material for: Development and characterization of a CRISPR/Cas9-mediated RAG1 knockout chicken model lacking mature B and T cells
Source: Front Immunol. 2022 Aug 11;13:892476. doi: 10.3389/fimmu.2022.892476 (PMC9403712; doi:10.3389/fimmu.2022.892476)
Supplement: Supplementary file 1 [file DataSheet_1.docx]

Supplementary Material

## Supplementary Figure 1


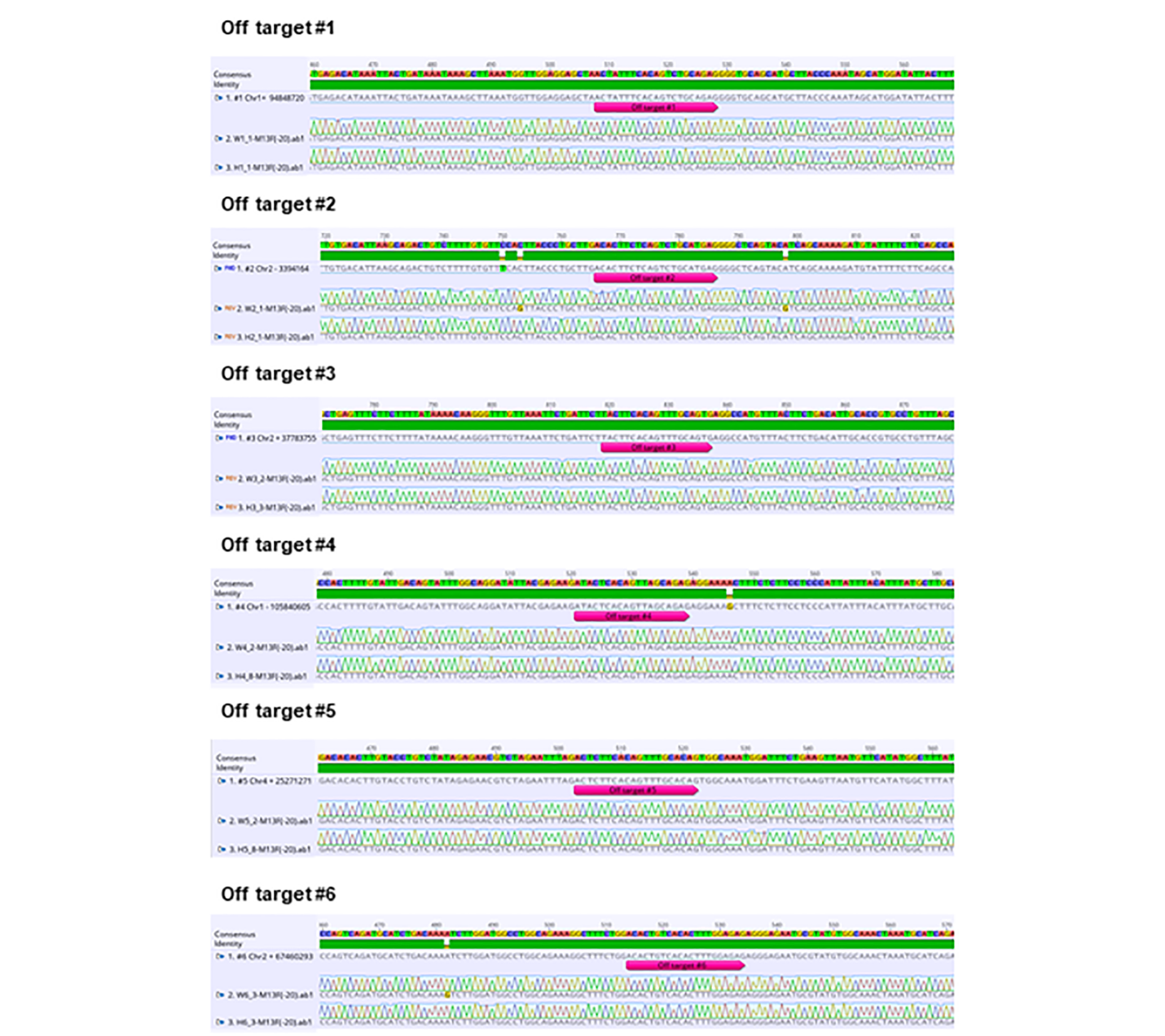


**Supplementary Figure 1.** Sanger sequencing chromatograms of putative off-target region in WT and RAG1 ^-/-^ chickens

## Supplementary Figure 2

##
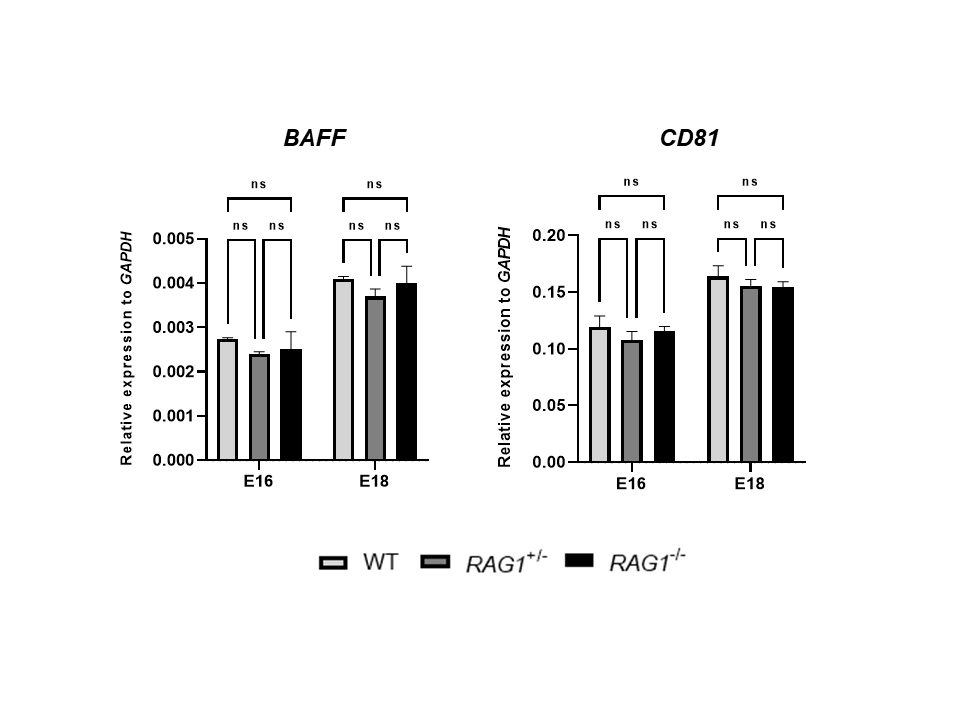


**Supplementary Figure 2.** Relative expression of B cell marker genes in E16 and E18 WT, *RAG1* ^+/-^ and *RAG1* ^-/-^ chickens bursa, calculated after normalization to *GAPDH*.

## Supplementary Figure 3

##
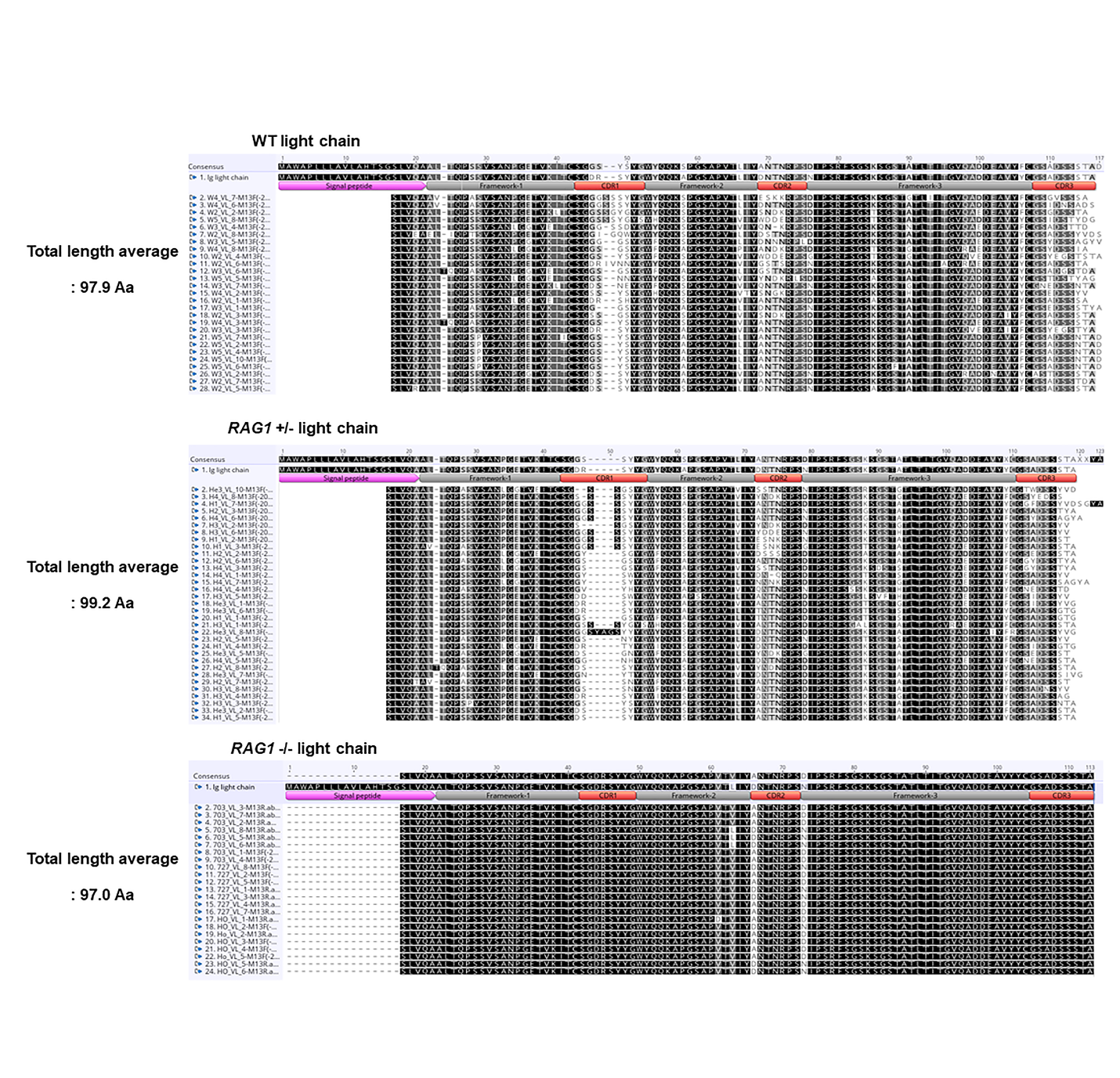


**Supplementary Figure 3.** Sequencing analysis of VJ rearrangement of the immunoglobulin light chains of PBMC gDNA isolated from 3-week-old WT, *RAG1* ^+/-^ and *RAG1* ^-/-^ chickens.

## Supplementary Figure 4

##
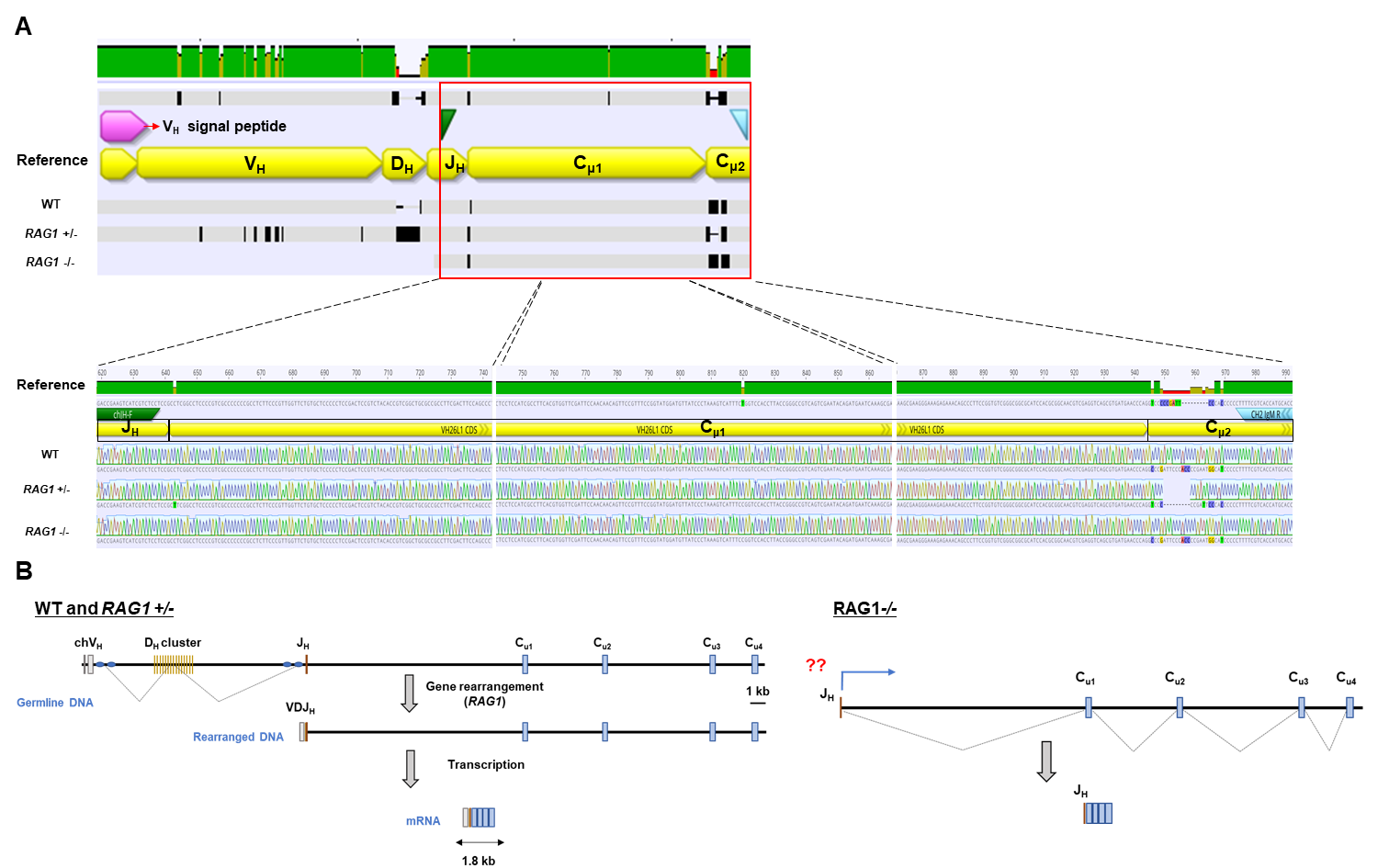


**Supplementary Figure 4.** Sequencing analysis of immunoglobulin (Ig) heavy chain transcript cDNAs from E18 of WT, *RAG1* ^+/-^ and *RAG1* ^-/-^ E18 bursa. (A) Entire heavy chain transcript was transcribed in WT and *RAG1* ^+/-^, while J segment to the constant region was transcribed in *RAG1* ^-/-^. (B) Schematic diagram of the expression of expected Ig in WT, *RAG1* ^+/-^ and *RAG1* ^-/-^.

## Supplementary Figure 5

##
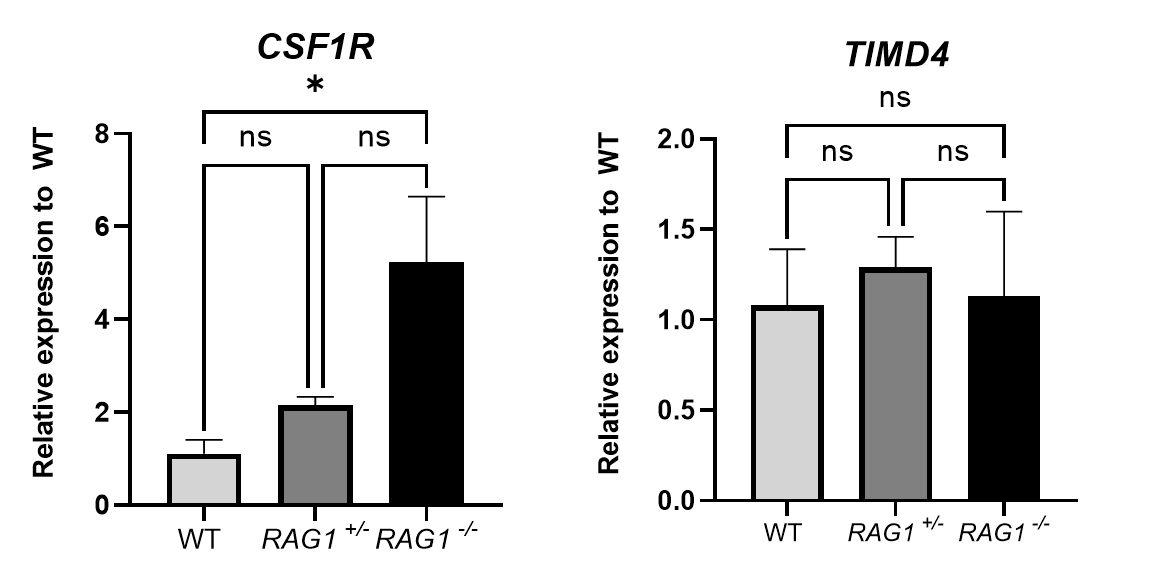


**Supplementary Figure 5.** Relative expression of macrophage marker genes in 3-week-old WT, *RAG1* ^+/-^ and *RAG1* ^-/-^ chickens bursa, calculated after normalization to *GAPDH* and WT samples. The significance of differences among groups was assessed by one-way ANOVA; * *P* < 0.05

## Supplementary Figure 6

##
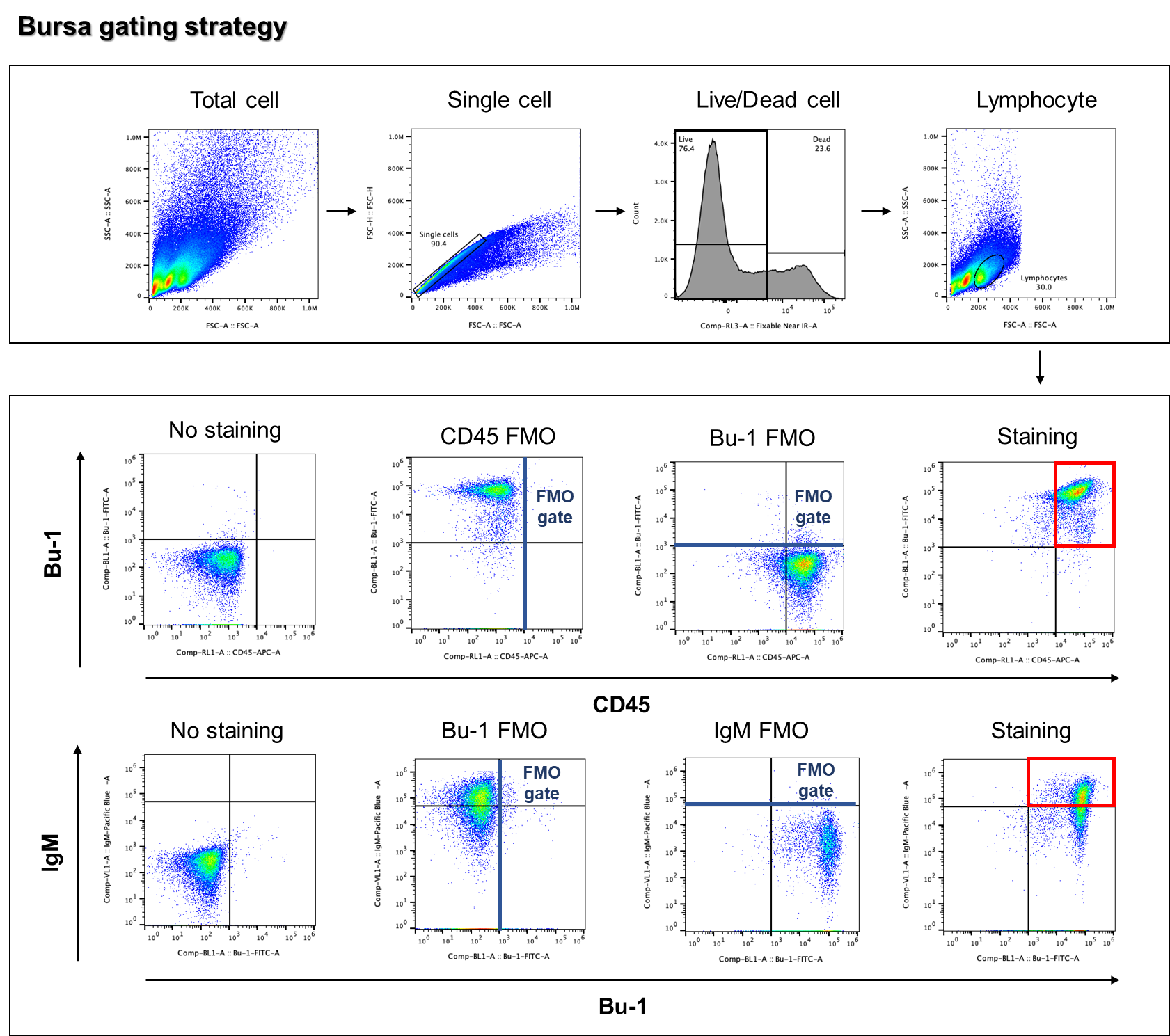


**Supplementary Figure 6.** Gating strategy to identify 3-week-old chicken Bu-1 and IgM positive cells in bursa.

Supplementary Figure 7

##
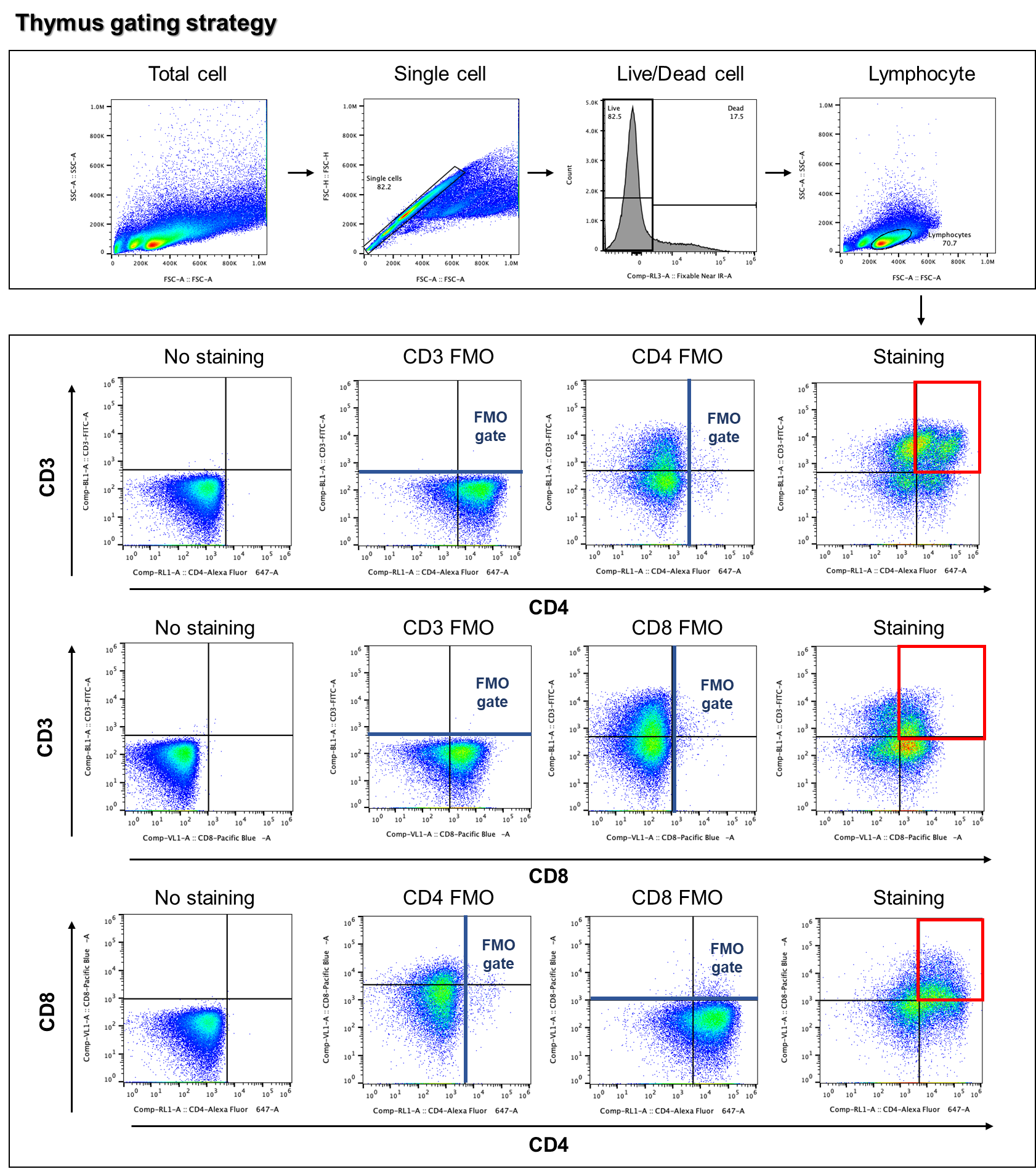


**Supplementary Figure 7.** Gating strategy to identify 3-week-old chicken CD3, CD4 and CD8 positive cells in thymus.**Supplementary Table 1. Oligos used in this study**

| **Oligo ID** | **Sequence (5’-3’)** | **Use** |
| --- | --- | --- |
| RAG1 #2 F | CACCGCTCTGCAAACTGTGAAGTGTG | *RAG1* #2 CRISPR/Cas9 vector construction |
| RAG1 #2 R | AAACACACTTCACAGTTTGCAGAGC |  |
| RAG1 seqF | AAGCAACTTTGCCGCATCTG | Genomic DNA PCR |
| RAG1 seqR | ACACGTTGTTTCCACTGGGT |  |
| RAG1 donor seqF | GGAAGAAAGCGAAAGGAGCG |  |
| RAG1 donor seqR | TATGCGAAGTGGACCTGGGA |  |
| VH-F | CACCAGTCGGCTCCGCAACCATG | Ig rearrangement PCR |
| JC-R | GCCCAAAATGGCCCCAAAAC |  |
| VL-F | GGCCGTCACTGATTGCCGTTT |  |
| VL-R | AGCCTGCCGCCAAGTCCAAG |  |
| VH F | GCTCCGTCAGCGCTCTCTGTCCTTC | IgM segment RT-PCR |
| DH F | GGTTACTGTGGTTGGAGTGCT |  |
| JH F | ATGGGGCCACGGGACCGAA |  |
| CH1-F | CAGTTCCGTTTCCGGTATGG |  |
| CH2-R | GGGGTGCATGGTGACGAAAAG |  |
| CH2-F | GGAGATGAGGAACACCAGCA |  |
| CH3-R | TTCTCCTTCCACCACGACAC |  |
| CH3-F | AGAGGGTCCTGCAAAGCAAC |  |
| CH4-R | CCGATGTGACCGATTGGCTT |  |
| RAG1 off target #1 F | GGGGATACAAGTCTATTTTGGGT | RAG1 off-target analysis |
| RAG1 off target #1 R | AACCCTCTGAGTCATGCTACA |  |
| RAG1 off target #2 F | GGCAGTGTATCACCTCTTCAGT |  |
| RAG1 off target #2 R | AACGAGGAGAACACGAGCTC |  |
| RAG1 off target #3 F | GCCAAAAGGCCGTCTTAGGA |  |
| RAG1 off target #3 R | CAACTGTGATCGCATGGAAGG |  |
| RAG1 off target #4 F | AAACCTCTGTGGGCTTCCTG |  |
| RAG1 off target #4 R | ATGAAGCACGACGTGTAGCT |  |
| RAG1 off target #5 F | GCTTGAGCAGCTTGTGTTGT |  |
| RAG1 off target #5 R | TCATAGCTTCAAGTCTGCCCTC |  |
| RAG1 off target #6 F | GTTGTGCCTGGCAGCTACTT |  |
| RAG1 off target #6 R | AAGGCTTTCCTCAGTATCCCAC |  |

**Supplementary Table 2. Oligos used for qRT-PCR analysis**

| **Oligo ID** | **Sequence (5’-3’)** | **Use** |
| --- | --- | --- |
| qRT RAG1 F | GAACAGCACGATGGAGTGGC | qRT-PCR analysis |
| qRT RAG1 R | GGTGCTGAGATGTCTGTTCTTG |  |
| qRT Ig Light F | CTTTGACGGTGGGAAGAGGG |  |
| qRT Ig light R | CGACGACGAGGCTGTCTATT |  |
| qRT BAFF F | CACGTCATCCAGCAGAAGGAT |  |
| qRT BAFF R | ACAAGAGGACAGGAGCATTGC |  |
| qRT Ig alpha F | CACCTATGAGGACGTGGGGA |  |
| qRT Ig alpha R | GCAAACCCCAAACAGCAGCG |  |
| qRT Ig beta F | GACAGCAAGAACCTCACGGA |  |
| qRT Ig beta R | GGTCTTCCTCAGGCCTTTCC |  |
| qRT Ikaros F | GTGCTCATGGTTCACAATCGAA |  |
| qRT Ikaros R | TTCACCCGAGTGCAACTTGA |  |
| qRT Pax5 F | CCACACCCAAAGTTGTCGAA |  |
| qRT Pax5 R | TGGGCACGGTGTCGTTATC |  |
| qRT AICDA F | GGTTGCCATGTGGAGGTTCT |  |
| qRT AICDA R | GTTTGGGTAGGCACGAAGGA |  |
| qRT CXCR4 F | CACAGAAGCCCTTGCGTTCT |  |
| qRT CXCR4 R | AGGCTTGATCCTCTGCTAACAGA |  |
| qRT CXCL12 F | ATCCCAAGCTAAAATGGATCCA |  |
| qRT CXCL12 R | AGCCCTTAACGTTCTACCCTTGA |  |
| qRT BAFFR F | CCTGGCCCCACCATAAGG |  |
| qRT BAFFR R | CATTACAGTCTCTCCTCACCCATACA |  |
| qRT NF-κB F | GAGGATGCTTCGTTGTGCTG |  |
| qRT NF-κB R | GCAGCATCCTCACATCTCCA |  |
| qRT LYN F | GAGAGCAGCTAATGTTCTGG |  |
| qRT LYN R | TCCATAGTTGATCGCCTCCG |  |
| qRT SYK F | CACCGTATCCTCATTGGGTCA |  |
| qRT SYK R | TGGTATGGAGAGCTTTCCTGTC |  |
| qRT PLCG2 F | GACAGCTGACAAGATCGAAGG |  |
| qRT PLCG2 R | TTGAGGACAAACTGGGTACCA |  |
| qRT BTK F | AAGAAGCGCCTGTTTCTGCT |  |
| qRT BTK R | AGGGGGAGGGTTGTTTTCAG |  |
| qRT CSF1R F | CCACTGCTTTTGGACTGGGA |  |
| qRT CSF1R R | GGTTAACAATGTTCGCGTGGT |  |
| qRT TIMD4 F | GAACCTGTGTTCTGCGCAAC |  |
| qRT TIMD4 R | TGAAGTGTGGTTGGCTCTGG |  |
| qRT GAPDH F | GGTGGTGCTAAGCGTGTTAT |  |
| qRT GAPDH R | ACCTCTGCCATCTCTCCACA |  |
